# Reply: Broca’s area: why was neurosurgery neglected for so long when seeking to re-establish the scientific truth? *and* Where is the speech production area? Evidence from direct cortical electrical stimulation mapping

**DOI:** 10.1093/brain/awab177

**Published:** 2021-05-12

**Authors:** Diego L Lorca-Puls, Andrea Gajardo-Vidal, David W Green, Cathy J Price

**Affiliations:** 1 Wellcome Centre for Human Neuroimaging, UCL Queen Square Institute of Neurology, London, UK; 2 Department of Speech, Language and Hearing Sciences, Faculty of Medicine, Universidad de Concepcion, Concepcion, Chile; 3 Faculty of Health Sciences, Universidad del Desarrollo, Concepcion, Chile; 4 Department of Experimental Psychology, University College London, London, UK

Gajardo-Vidal *et al*.^1^ reported data from a sample of 134 stroke patients with relatively circumscribed damage to the left frontal lobe. Contrary to the received view, mapping lesion to deficit using an ecologically-valid measure of speech production (i.e. spontaneous connected speech elicited with a picture description task) revealed that, irrespective of lesion extent, damage to Broca’s area does not contribute to long-term speech production outcome, whereas damage to the white matter in the vicinity of the anterior part of the arcuate fasciculus (AF) is strongly implicated. Our results therefore address a matter of critical clinical importance: understanding the causes of inter-patient variability in speech production outcome post-stroke. 

We welcome the opportunity to reply to two Letters to the Editor on our paper.^2^^,^^3^ Both Letters rely on a source of data different from ours: direct electrical stimulation (DES) and/or resection carried out during neurosurgical procedures on awake patients (e.g. with glioma). A key behavioural outcome measure in such work is the presence or absence of speech arrest (e.g. an inability to name a visually presented picture) following DES over a given cortical region. Inferences drawn from different clinical populations and methodological approaches that converge on a common scientific understanding (consilience) bolster confidence in that understanding, and so we strongly endorse the contribution of research in neurosurgical patients to the existing body of knowledge regarding the regions involved in speech production. Consilience is important because different methods have different strengths and weaknesses. For instance, the challenge for lesion-deficit mapping research in stroke patients is to dissociate the contribution of brain regions that are commonly damaged together. In the case of DES, an observed behavioural effect is not necessarily the consequence of direct stimulation of the targeted region; it may instead reflect the contribution of stimulation that spreads along white matter pathways into remote regions.^4^ In the case of resection (and DES), the absence of a behavioural effect might reflect functional reorganization that has occurred overtime prior to neurosurgery, due to an underlying neurological condition (e.g. tumour or epilepsy). This contrasts with the sudden onset of stroke where functional reorganization is not expected to occur until after the insult.

In what follows, we identify the main contentions of each Letter first and then address them one by one.

Mandonnet and Duffau^2^ propose that prior data, acquired in patients undergoing neurosurgery, already refute the relevance of Broca’s area to persistent speech production impairments. They highlight, for example, the work of Benzagmout *et al*.^5^ where focal resection of Broca’s area only resulted in transient speech production impairments. They also enjoin the community to understand the apparent neglect of such data. We recognize the perpetuation of the myth of Broca’s area as the critical region for speech production, but challenge the decisiveness of current neurosurgical data with respect to the research question we addressed. For example, the Benzamount *et al*.^5^ data may establish that damage to Broca’s area is not sufficient to cause long-lasting speech production impairments, but they do not refute its necessity in combination with surrounding regions (including the underlying white matter), which was the focus of our work.

Zhao *et al*.,^3^ based on their work with glioma patients, offer convergent evidence that the anterior segment of AF is essential and conclude that this region is the speech production area. We reprise our arguments about the anterior part of AF for the sake of clarity, but strongly contest the localizationist notion that the anterior segment of AF is the speech production area. We emphasize, instead, the critical importance of understanding the network of regions involved in speech production.

Madonnet and Duffau^2^ set their comments in an historical context. We also set our findings from stroke patients in this context. For example, our results refine the influential work of Mohr *et al*.^6^ These authors were the first to systematically show that relatively focal damage to Broca’s area typically causes an initial mutism that rapidly evolves into mild speech production impairments or completely resolves over time. Moreover, Mohr *et al*.^6^ showed that the severe and persistent speech production impairments originally described by Paul Broca are associated with far more extensive damage that involves Broca’s area and neighbouring brain regions. These findings led to the long-held belief that persistent speech production impairments after damage in and around Broca’s area are caused by the combination of damage to Broca’s area and neighbouring regions. We showed, however, that damage to Broca’s area does not contribute to long-term speech production outcome, irrespective of the extent of the lesion. Therefore, our study’s findings complement and extend those of Benzagmout *et al*.^5^

DES (and resection) data provide evidence for the role of a cortical region or white matter tract in speech production and we cited pertinent work, and reviews of the topic, in our paper. Speech arrest, however, does not allow immediate inference to the long-term consequences of damage to an area. Moreover, the evidence for speech arrest with DES of Broca’s area has been inconsistent. We placed most weight on data from Tate *et al*.^7^ involving a large sample of 165 neurosurgical patients that indicated only rare instances of speech arrest. But still, as a community we need to acknowledge methodological factors associated with the use of DES. For instance, the absence of an effect may arise because stimulation intensity is insufficient,^8^ because of prior functional reorganization in response to tumour infiltration/epileptic foci, or because the task assessing speech arrest (e.g. picture naming) is not sensitive enough to the function of the region (e.g. sentence generation).

If damage to Broca’s area does not contribute to long-lasting speech production impairments, what may explain such impairments in stroke survivors with damage in and around Broca’s area? Prior DES/resection data leave this question open. Our analyses indicated a causal role for co-occurring white matter damage in the vicinity of the anterior part of AF and established that such an outcome is not a consequence of the disconnection of Broca’s area. In brief, we found that the speech production abilities of patients with direct damage to Broca’s area and relative sparing of the white matter in the vicinity of the anterior part of AF were, on average, within the normal range and significantly better than those of patients with the contrasting pattern of damage (i.e. to the white matter in the vicinity of the anterior part of AF with relative sparing of Broca’s area). Thus, our data establish that damage to Broca’s area is neither necessary nor sufficient to cause persistent speech production impairments.

Interestingly, Zhao *et al*.^3^ report DES data on 18 glioma patients bearing on the role of the anterior and long segments of AF in speech production. In our study, we noted high collinearity in the degree of damage to these two segments, which precluded us from dissociating their effects. Zhao *et al*.^3^ report a dissociation: the frontal end points of the anterior segment are more consistent in eliciting speech arrest. We look forward to a detailed report, as such data would exemplify the value of consilience. In the meanwhile, we remain cautious as their Letter suggests that they stimulated the frontal cortex where these two AF segments are thought to terminate based on tractography data (yielding indirect evidence), or perhaps stimulated the fibres directly after resection of the corresponding cortex (potentially conflating resection and stimulation effects).

On their claim that the frontal termination of the anterior segment of AF is the speech production area, we disagree with such a localizationist position. In our paper, we stated that the long-lasting detrimental effect of damage to the white matter in the vicinity of the anterior part of AF ‘may be the consequence of disrupted functional integration among the multiple regions in inferior frontal, inferior parietal and superior temporal cortices involved in the sensorimotor control of speech production … irrespective of whether or not Broca’s area has been disconnected’.^3^ This is consistent with the results reported by Herbet *et al*.,^9^ based on DES and tumour resection data, suggesting that the compensatory potential is generally high after cortical damage and low after white matter damage (particularly for AF).

Where is the speech production area? Our view is that no such area is discoverable by DES, or any other method for that matter, because no such area exists. Speech production results from the coordinated activity of multiple brain regions, in which the network connectivity of AF may be salient.

##  

### Data availability

Data availability is not applicable to this article as no new data were created or analysed in this study.

## Funding

This work was funded by Wellcome (203147/Z/16/Z and 205103/Z/16/Z, C.J.P.), the Medical Research Council (MR/M023672/1, C.J.P.) and the Stroke Association (TSA 2014/02, C.J.P. and D.W.G.).

## Competing interests

The authors report no competing interests.
