# Erratum to: Reply: Broca’s area: why was neurosurgery neglected for so long when seeking to re-establish the scientific truth? *and* Where is the speech production area? Evidence from direct cortical electrical stimulation mapping

**DOI:** 10.1093/brain/awab229

**Published:** 2021-07-19

**Authors:** Diego L Lorca-Puls, Andrea Gajardo-Vidal, David W Green, Cathy J Price

Diego L. Lorca-Puls, Andrea Gajardo-Vidal, David W. Green, Cathy J. Price. Reply: Broca’s area: why was neurosurgery neglected for so long when seeking to re-establish the scientific truth? *and* Where is the speech production area? Evidence from direct cortical electrical stimulation mapping. Brain 2021;awab177. doi:10.1093/brain/awab177

The publishers apologize for an error in the title of the article. This has been corrected.

